# Whose problem is it anyway? Crimes against women in India

**DOI:** 10.3402/gha.v7.23718

**Published:** 2014-07-21

**Authors:** B. L. Himabindu, Radhika Arora, N. S. Prashanth

**Affiliations:** 1 Institute of Public Health, Bangalore, India; 2 Institute of Tropical Medicine, Antwerp, Belgium

**Keywords:** Delhi gang rape, women in India, child sex ratio, gender inequality

## Abstract

The recent public outcry following a brutal gang rape of a young woman in India's national capital was a watershed moment in the world's largest democracy. It generated widespread public and political support for strengthening legal provisions to punish sex offenders. Although the legal response is a useful deterrent against such heinous crimes, women continue to suffer due to deeply rooted social prejudices that make them vulnerable to violence and discrimination in society. In this commentary, we aim to analyse the current developments with respect to gender violence in India within a background of the social position of women in Indian society. Using secondary data related to sex-selective abortions and crimes against women, and a critical review of the portrayal of women in Indian cinema, we reflect on the role of health workers, researchers and public health professionals in shaping a social response towards improving gender parity in our country.

On 13 September 2013, a New Delhi judge sentenced four men to death for the brutal gang rape of a 23-year-old physiotherapy student. She died due to severe injuries suffered during the attack. The barbaric nature of the crime appalled the country and brought worldwide attention to what print media now calls the *Rape crisis* of India (1, 2). Nationwide protests forced lawmakers to refer this case to a fast-track court, and the judgment was pronounced in less than a year. The perpetrators were sentenced to the gallows, a punishment reserved in Indian law for the *rarest of the rare* instances of inhuman crime. The government sought to appease the widespread street protests in many cities. The law concerning violence against women was amended by the Parliament (3). The maximum punishment for rape resulting in death (or vegetative state) of the victim was modified from life imprisonment to include death penalty. Other laws related to sexual crime were made stricter, in the hope that this would deter people from committing such crimes. The source and subsequent impact of these immediate reactions and quick fixes have been extensively debated in the print media (4, 5).

In this commentary, we aim to discuss the current scenario of violent crimes against women in India within a backdrop of the social position of women in Indian society in general and the media response following this incident in particular.

## Crimes against women in India

The numbers of violent crimes in India especially those against women including rape that are reported in official statistics are increasing with each passing year (6). This violence thrives within a milieu of steady economic growth, and increasing inequality between the rich and poor in Indian society; India's GINI coefficient^1^ that has increased from 0.32 to 0.38 in the last two decades is evidence to that (7). India's new riches and development strides as witnessed by its GDP growth from $450.42 billion in 2000 to $1841.7 billion seem to bear no fruits for its women. In 2012, the crimes against women reported by official statistics increased by 24.7%, compared to those reported in 2008 (8). Ranging from the so-called *eve teasing*^2^ and outright sexual harassment on the street or workplace, to harassment for *dowry*,^3^ molestation in public transport vehicles, and the often-reported rape, these crimes against women reflect the vulnerability and deep-rooted problems related to the position of women in Indian society. Out of 28 states, 10 states reported more than 10,000 cases of crime against women in 2011 putting states with both high and low HDI (Human Development Index) and literacy rates in the list; probably an indication that education and economic growth alone do not influence the occurrence of these crimes and pointing towards socio-political and cultural factors. This can be further observed in the National Crime Records Bureau (NRCB) statistics which show that cruelty by a husband or his relatives (46.8%) and *dowry*-related crimes (7.1%) account for more than half of the crimes against women. With increased incidence and visibility of these gruesome crimes, there is an urgent need to address this problem at multiple levels of Indian society, including professional, familial and social settings.

According to the NCRB, 24,923 cases of rape were reported in 2012 (9), amounting to one rape every 22 minutes. A continuous increase in the reported cases of rape has been observed in the period from 2009 to 2012 with more than 3% increase in the number of cases reported in 2012 over 2011. Nearly, 12.5% (3,125) of the total victims of rape were girls younger than 14 years, 23.9% (5,957) were in the 14–18 age group, 50.2% (12,511) were in the 18–30 age group and 12.8% (3,187 victims) were in the 30–50 age group. These statistics possibly do not capture the actual numbers. While gross under-reporting could be one reason for this (10), the other reason is that crimes such as gang rapes, stalking and acid attacks on women were not included in official statistics of crime against women until the law was amended on 3 February 2013 (3). Even amongst those crimes, the NCRB statistics takes only the principal offence of the formal complaint (First Information Report) into account. So in cases such as the Delhi gang rape, which resulted in the death of the rape victim, the rape would be unaccounted for in the official statistics; the true scale of gender violence thus remaining undercounted.

The problem of underestimation of the gender-based crime is compounded by failure of the justice system of the country in securing convictions. The NCRB statistics (9) show that 54.6% of rape cases reported in 2011 are yet to be investigated, while 30.6% are waiting for trial. Only 16% of the cases have resulted in convictions. The recent protests demanded for stricter laws, possibly under an assumption that greater punishment will reduce the rate of sexual crime. However, with the abysmal conviction rate, stricter laws alone may not achieve the necessary deterrence.

The amendments made to the criminal law (3) are not (yet) comprehensive. Marital rape, for example, is still not considered a criminal offence. Rape by armed personnel (military and police), although under the purview of the law, is excluded if it occurs in several states of India (north-eastern states, Jharkhand, and Jammu and Kashmir) where the draconian Armed Forces Special Powers Act (AFSPA) deprives women from seeking legal recourse in such circumstances. The consequences of having separate law for Armed forces are many. For example, the alleged mass rape of 53 women in 1991 and the alleged rape and subsequent killing of Manorama Devi in the year 2000, both incidents involved armed forces personnel. Both cases are still in court and the verdict is yet to be delivered, decades after the crimes (11, 12).

On one hand is the political apathy in formulating and implementing gender-sensitive policies. On the other is the lack of a clear protocol of action in these issues, care of victims of rape being one such example. Although a detailed directive was sent to all the state governments on establishment of Rape Crisis Centres (RCCs) and specialised Sexual Assault Treatment Units (SATUs) in 2009, no such units have been set up in the states even now, except in New Delhi (13). The law states that a female police officer should record the victim's statement, as well as assist her with medical and legal support. However, female police personnel account for only 6.5% of the police force, which makes it difficult to implement this. Further, the government health services in the country lack the infrastructure and resources needed to implement care for rape victims as specified by the law in most district and sub-district hospitals. It has been reported that traumatised victims often have to go from one hospital to another for forensic examination following rape (14). Victims often sit for hours in soiled clothes in the hospital and feel humiliated all over again in the course of insensitive history-taking by doctors and health workers. Judgemental attitudes and lack of privacy in government healthcare establishment worsens their trauma (14).

## Tip of an iceberg: women in Indian society

In a democracy, it is said that the politicians are only as good as the people. The deep-rooted patriarchy of Indian society lay exposed when several people, including senior politicians, type casted the victims of sexual violence, as possibly having contributed to the perpetration of the crime (15). Some of the typical characterisations of the victims included women who dressed ‘provocatively’, ‘was out late in the night’ or was ‘behaving in a suggestive way that invited trouble’. Others suggested in an apparent gesture of sympathy that the rape victim becomes a *living corpse* indicating the life of shame that the victims of sexual abuse will be subjected to in the country.

The problem of gender-based violence runs very deep in India. The rape crisis is just one facet of the multitude of problems that reflect the gender discrimination scenario. These prejudicial attitudes are seen right from womb to tomb (16). They start with the practice of sex-selective abortion and infanticide, and continue through adolescent and adult life with high levels of female infant mortality, child marriage, teenage pregnancy, lesser wages for women, unsafe workplaces, domestic violence, maternal mortality, sexual assault and neglect of elderly women.

India has made great strides in terms of economic growth in the past decade. The increase in female literacy rates from 54% in 2001 to 65% in 2011 (17) and improved maternal mortality ratio from 327 in 2001 (18) to 178 in 2012 (19) suggest a movement towards greater gender equality in the country. However, the 2011 census showed that the child sex ratio dropped to its lowest value since independence to 914 females for every 1,000 males, showcasing the continuing trend of boy preference. What is surprising is that socio-economic development alone does not modify this trend. Even states that rank high on the HDI report relatively low child sex ratio and high female infant mortality rate (see Table 1) (20–22). In other aspects of gender equality too, such as education or participation of women in the workforce, or representation of women in elected bodies, India falls short of international standards. It ranks 136 out of 186 among the nations of the world in the United Nations Development Programme's gender inequality index. Violence against women, either sexual or physical – wife beating is widely spoken of, but hardly reported – is an expression of power asymmetry between men and women. It seems to co-occur with other indicators of gender disparity. The dismal reporting rate of domestic violence can also be attributed to the attitudes of the victims. According to National Family Health Survey 3 (NFHS-3) data, 41% of women believed their husbands were justified in slapping them and 35% of the women even believed that a brutal beating is also justified if they neglected doing the household chores or looking after their children.

**Table 1:** Comparison of the CSR and FIMR amongst Indian states with High and Low Human Development Index

State	Human Development Index	CSR (the number of females per thousand males in the age group 0–6 in a human population)	FIMR (the number of deaths of female children less than one year of age per 1,000 live births)
India	0.554	914	52
Delhi	0.750 (Rank=3)	866	34
Himachal Pradesh	0.652 (Rank=4)	906	45
Punjab	0.605 (Rank=5)	846	39
Haryana	0.552 (Rank=9)	830	53
Odisha	0.362 (Rank=22)	934	66
Chhattisgarh	0.358 (Rank=23)	964	57

*Sources*: From Refs. 20, 21, and 22.

CSR, child sex ratio; FIMR, female infant mortality rate.

## Women and Indian cinema

The portrayal of societal themes in popular cinema could be considered as a reflection of popular societal attitudes. In Indian cinema, ‘kissing’ was not allowed on-screen on the grounds of modesty until the mid-2000s. However, rape or *izzat lootna* (dishonouring) of women has been a recurrent theme and sub-theme in mainstream Bollywood cinema for decades now, examples are movies like *Insaaf Ka Tarazu* or more recently *Woh Lamhe*. Rape and subsequently avenging rape often forms the central narrative of many films. Rape also appears as a sub-plot to reinforce the heroic role of male actors in films (23). The familiar portrayal of rape and sexual assault of women in cinema, however tacit, is disturbing in its lack of censorship (versus censorship of acts like kissing, for example) and its conflicting pervasiveness in a mainstream form of entertainment. However, this is not to deflect from the limited, but realistic representation of rape and forms of sexual abuse in alternate films such as *Bandit Queen* 4 (Shekhar Kapur, 1994) and *Monsoon Wedding* (Mira Nair, 2001) which used the film media to bring the issues into mainstream discourse. Another genre of mainstream media the *saas–bahu serials* (translation: *mother-in-law and daughter-in-law* soaps) have been acknowledged for their role in featuring other forms of violence and discrimination within Indian households. At a time when the country is introspecting its treatment of women, it would be useful to remind ourselves that sexual violence in the popular media may be a way of highlighting issues of violence against women and may also, in many cases, be an echo of pervasive prejudices in our society (24). Although the presence and acceptance of violence against women in mainstream media in India warrants further research, its existence as a reflection of societal attitudes is indeed indisputable (Box 1).

Box 1 **Women and Indian cinema**

In the 1978 film *Ghar*, a newlywed couple on their way home, are attacked by a group of young men (drinking, driving a car and listening to music). The wife is abducted and raped. The rest of the film/script aside, the construction of the 3 min sequence from 1978 could be considered reflective of society even today. The point to note here is the portrayal of the woman. She is portrayed as an honourable or a virtuous woman owing to the fact that she was (a) married; (b) she was walking with *her husband* when she was attacked. The rapists’ portrayal however was as men not known to the victim, but rather as indulgent young men out to have a good time in a car, under the influence of alcohol. According to 2012 NCRB data, rapists were known to the victim in as many as 98.2% of the cases (15).

While on one hand it is essential to develop comprehensive laws to address this issue, laws are not the only solution. The effectiveness of these laws depends on women's awareness of these laws and their ability and ease to call upon them, if need be. Legal awareness among women is an important step towards improving the reporting rates of rapes. Allotment of adequate funds to build the necessary infrastructure and to ensure the enactment of this law through qualified and trained personnel are the steps to be taken by the government. In terms of judicial measures, although the amendment to criminal law states that all rape cases should be tried in fast-track courts and the trial to be completed within 2 months, without the necessary judicial reforms and infrastructure in place it will be extremely difficult to achieve the desired conviction rate. According to NCRB statistics, 83.6% of cases are still pending in courts across various states in the country. With this poor conviction rate, even the most stringent of laws do not serve their purpose.

The government of India failed to incorporate several important recommendations of the three- member committee that was formed to amend the criminal law (3), the crucial one being the criminalisation of marital sexual abuse. According to the National Family Health Survey report of 2005–06 (25), 9% of all women aged 15–49 experienced sexual violence at some time during their lifetime. Out of these 87.5% reported that the perpetuator was their current husband. With over 104 countries in the world outlawing marital rape, it is only imperative for India to follow in their footsteps without using tradition or the institution of marriage as an excuse.

## Gender-based violence and public health

As health workers (doctors, nurses and public health professionals), we all come across various facets of this issue. Be it an out-patient encounter with a victim of domestic violence presenting with non-specific complaints, or post-rape care of a victim at a primary care facility, health workers’ appreciation of this societal problem is an important part of the solution. Public health implications of rape are numerous. The repercussions of sexual violence are beyond the victim and include the family as well as society at large.

According to the NCRB statistics (6), out of the 24,923 reported rape cases in the country during the year 2012, offenders were known to the victims in as many as in 24,470 (98.2%) cases. Primary healthcare/emergency healthcare professionals, who may be the first point of contact for these victims should be able to recognise the signs of a sexual assault and report them to the appropriate authorities. It is essential to train these personnel not only in medical treatment of these victims, but also in providing psychosocial support to them. To incorporate routine screening of violence into healthcare practise, healthcare professionals’ preparedness to treat such patients needs to be assessed and they should be trained in the maintenance of confidentiality, positive attitudes, and respect for patients’ rights.

Provision of medical as well as legal support to the victims at their first point of contact following the incident can increase the reporting as well as conviction rate in these crimes. Ensuring the provision or providing the victims with emergency contraception pill (ECP) or post-exposure prophylaxis (PEP) should be the responsibility of the first point of contact as these are immediate measures, most effective within the first 72 hours after the incident. Development and training of both police as well as medical personnel on standardised protocols in post-rape care is imperative.

Conviction in any criminal case largely depends on the forensic evidence. Abysmal conviction rate of rape cases, standing at 16% of the total, can be improved by meticulous forensic sample collection and transfer to the concerned authorities. Every medical officer in public health services should be trained to perform physical assessment as well as meticulously maintaining the medical records. They should be trained in sample collections of forensic evidence from the victims and aid the police and other concerned authorities in providing evidence. Advocacy campaigns about the optimum post-rape care should be undertaken by public health professionals to achieve these goals.

Establishment of RCCs and SATUs in all major cities and towns is essential, which can act as a one-stop centre for medical, social, psychological and legal support of rape victims. Government, in collaboration with NGOs and other public health organisations, should set up helplines through which the victims have access to a network of professionals who are trained to support them in seeking care as well as legal recourse. Gender sensitisation of personnel towards this sensitive issue is another step that needs to be taken to make sure that the law enforcers as well as medical staff – largely men – are more sensitive towards rape victims.

## Conclusion

Gender-based violence, especially violent crime like rape, is a multifaceted problem. To address this, it is essential to tackle various other concurrent issues that act as contributing factors and thus play an equally important role. An example for this is the portrayal of women in Indian cinema. This bears evidence to the deep-rooted prejudicial attitudes towards women and other deeper societal issues that are contributory to these crimes. Although the incorporation of stringent laws and stricter punishments are important to deter people from committing such crimes, the solution to this is much more than just promulgation. Though the amendment to criminal law addresses a few of these issues, it still falls short in many aspects. It is important to acknowledge that judicial reform is only one aspect; there is a more humane side to this whole issue. Legal solutions in the form of amendments to improve conviction rates could function as deterrents to such acts. However, in such a scenario health workers could play a key role in applying a gender lens to their work as healthcare providers, researchers and policymakers. In a country with gender discrimination operating at so many levels and in so many ways, bringing about the needed change requires dedicated and combined efforts of multiple agencies. While education and empowerment of women is a larger social process to which public health professionals may not be able to contribute directly, we urge health workers and public health professionals to facilitate improved access, utilisation and coverage of women in the services that we study, plan, implement and evaluate. Doctors, nurses and other healthcare providers, researchers and public health professionals need to respond to this social predicament individually and engage with this problem in their own families, organisations and communities.

## Footnotes

1 GINI coefficient: The GINI coefficient is a measure of statistical dispersion intended to represent the income distribution of a nation's residents.

## Footnotes

2 Eve-teasing: the making of unwanted sexual remarks or advances by a man to a woman in a public place.

## Footnotes

3 Dowry: an amount of property or money brought by a bride to her husband on their marriage.

## Footnotes

4 Bandit Queen is based on a real-life bandit turned politician, Phoolan Devi.
